# Metabolic engineering of *Escherichia coli* for the production of butyric acid at high titer and productivity

**DOI:** 10.1186/s13068-019-1408-9

**Published:** 2019-03-22

**Authors:** Liang Wang, Diane Chauliac, Brelan E. Moritz, Guimin Zhang, Lonnie O. Ingram, K. T. Shanmugam

**Affiliations:** 1 Department of Microbiology and Cell Science, University of Florida, Gainesville, FL 32611 USA; 2 State Key Laboratory of Biocatalysis and Enzyme Engineering, College of Life Science, Hubei University, Wuhan, 430062 China; 3 Present Address: Galactic, Brussels, Belgium

**Keywords:** Butyrate, Fermentation, *E. coli*, Xylose, Mineral salt medium

## Abstract

**Background:**

Several anaerobic bacteria produce butyric acid, a commodity chemical with use in chemical, pharmaceutical, food and feed industries, using complex media with acetate as a co-product. Butyrate titer of various recombinant *Escherichia coli* did not exceed 10 g l^−1^ in batch fermentations in any of the media tested.

**Results:**

A recombinant *E. coli* (strain LW393) that produced butyrate as the major fermentation product was constructed with genes from *E. coli*, *Clostridium acetobutylicum* and *Treponema denticola*. Strain LW393 produced 323 ± 6 mM (28.4 ± 0.4 g l^−1^) butyric acid in batch fermentations in mineral salt medium with glucose as C source at a yield of 0.37 ± 0.01 g (g glucose consumed)^−1^. Butyrate accounted for 90% of the total products produced by the culture. Supplementing this medium with yeast extract further increased butyric acid titer to 375 ± 4 mM. Average volumetric productivity of butyrate with xylose as C source was 0.89 ± 0.07 g l^−1^ h^−1^.

**Conclusions:**

The butyrate titer reported in this study is about 2.5–3-times higher than the values reported for other recombinant *E. coli* and this is achieved in mineral salt medium with an expectation of lower purification and production cost of butyrate.

**Electronic supplementary material:**

The online version of this article (10.1186/s13068-019-1408-9) contains supplementary material, which is available to authorized users.

## Background

Butyric acid is an aliphatic short-chain fatty acid with several industrial and pharmaceutical uses [1]. One of the major applications of butyric acid in chemical industry is in the production of cellulose acetate butyrate polymers. Butyric acid esters are common in the food and cosmetic industries as flavor and fragrant additives. Ethyl and butyl esters of butyric acid can directly serve as renewable fuel to mitigate petroleum use [2]. Further, butyric acid can be reduced chemically or biologically to butanol, a drop-in biofuel [3]. Role of butyric acid in human and animal health is being widely recognized and several attempts to increase colonic production by resident and/or introduced microorganisms are underway [4–6]. Butyrate has been reported to have anticancer activity and is rapidly becoming a feed supplement to improve animal health [7]. Dominant use of butyric acid is as a feed supplement followed by its use in the chemical industry and demand for this chemical is projected to grow by about 14% per year during the years 2015–2022 [8]. Most of the butyric acid is commercially produced today by oxidation of butyraldehyde derived from propylene from fossil fuels [1, 9]. There is an increasing preference for biologically derived butyric acid, especially in the animal feed market and for human use [1, 9]. Although butyric acid is a native fermentation product of several anaerobes, a bio-based butyrate industry is yet to develop and compete favorably with petroleum-derived butyric acid, probably due to the cost of production [9].

**Fig. 1:**
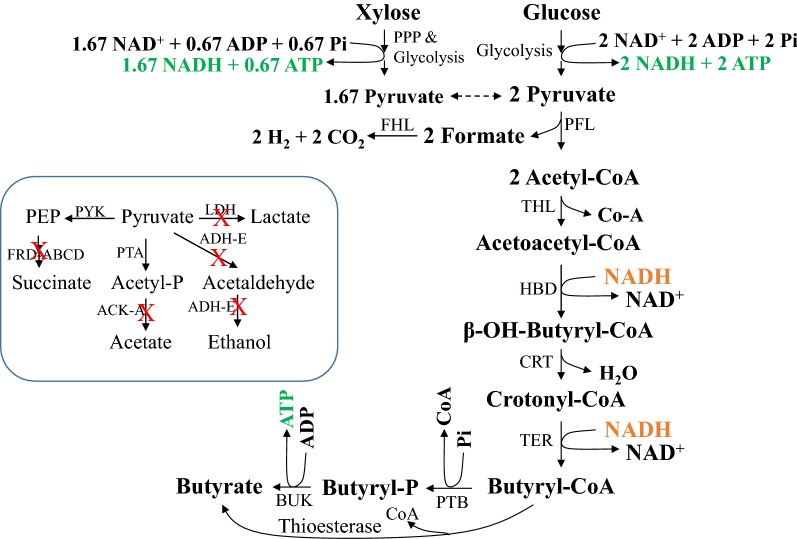
Butyrate pathway in the recombinant *E. coli* strains used in this study. In this proposed pathway, xylose is transported by the ABC transporter (XylFGH) and glucose is transported by its PTS system. PPP, pentose-phosphate pathway; PFL, pyruvate formate–lyase; FHL, formate hydrogen–lyase; THL, thiolase; HBD, hydroxybutyryl-CoA dehydrogenase; CRT, crotonase; TER, transenoyl-CoA reductase; PTB, phosphotransbutyrylase; BUK, butyrate kinase. HBD, CRT, PTB and BUK are from *C. acetobutylicum*. Ter is from *T. denticola*. Other enzymes are from *E. coli*. The native pathways of anaerobically growing *E. coli* at the pyruvate/phosphoenolpyruvate node and the mutations (marked by “X”) in strain BEM9 are listed in the boxed area. PFL, another native enzyme at the pyruvate node catalyzes the conversion of pyruvate to acetyl-CoA, the starting point of the butyrate pathway. PYK, pyruvate kinase; FRD-ABCD, fumarate reductase, the terminal enzyme of the PEP-succinate pathway; PTA, phosphotransacetylase; ACK-A, acetate kinase; ADH-E, acetaldehyde/alcohol dehydrogenase; LDH, D-lactate dehydrogenase

Clostridia ferment sugars to butyric acid as a component in a mixture of products [10]. Due to the need for redox balance during anaerobic growth, acetate is a major co-product of butyrate production by *Clostridium* spp. [11]. Butyrate is also an intermediate product of Clostridia that produce butanol. These bacteria, such as *C. acetobutylicum*, produce butyrate during growth that is later reduced to butanol during solventogenic phase [12]. Butanol, as an excellent drop-in biofuel, has the potential to replace ethanol in current blends of gasoline. However, the toxicity of butanol to the producing bacterium limits its titer and the highest titer in simple batch fermentations is less than 20 g l^−1^ [13]. Under similar conditions, the highest reported butyrate titer can exceed 45 g l^−1^ including moderate thermophiles like *C. thermobutyricum* [10, 14]. Fermentative production of butyrate at these titers coupled with a second-stage reduction, biologically or chemically, has the potential to generate higher concentrations of butanol than by direct fermentation of sugars to butanol. The reductant for this process can be the H_2_ produced by the same bacteria during butyrate production (Fig. 1).

To further increase the butyrate titer, metabolic engineering of native butyrate producers has been attempted [10, 15, 16]. Co-product of acetate in these fermentations is apparently due to the catalytic property of butyryl-CoA dehydrogenase (BCD) utilized by native Clostridia for the reduction of crotonyl-CoA to butyryl-CoA in the butyrate biosynthetic pathway [11]. For this two-electron/proton reduction step, BCD complex utilizes two NADHs resulting in the products butyryl-CoA and reduced ferredoxin. Reduced ferredoxin generated in this reaction is further converted to H_2_ by hydrogenase [17]. The redox imbalance created by this unique biochemical reaction is apparently a cause of acetate co-production by native Clostridia. Deletion of the hydrogenase in *C. acetobutylicum* increased the NADH pool and minimized acetate production [16].

A transenoyl-CoA reductase (Ter) from several organisms has been reported to reduce crotonyl-CoA to butyryl-CoA with only one NADH [18]. Substituting this enzyme for BCD would yield a redox balanced butyrate pathway (Fig. 1). Genes encoding various enzymes in the butyrate pathway with Ter (*ter*) substituting for BCD complex for crotonyl-CoA reduction to butyryl-CoA (Fig. 1) have been introduced into *Escherichia coli* and the highest butyrate titer of these recombinants in batch fermentations was about 10 g l^−1^ (Table 1) [19–26]. Some of these constructs utilized thioesterase(s) to remove the CoA from butyryl-CoA in the final step in butyrate production (Fig. 1) or a CoA transferase [19–26]. The main objective of this study is to metabolically engineer an *E. coli* for the production of butyrate utilizing phosphotransbutyrylase (PTB) and butyrate kinase (BUK) for catalyzing an ATP-yielding final step as in the clostridial butyrate pathway [27, 28], in mineral salt medium at a rate that is comparable to Clostridia. This contrasts with the complex medium required for growth and fermentation of sugars to butyrate by native producers and further helps to reduce the overall process cost.

**Table 1:** Comparison of fermentation characteristics of butyrate-producing *E. coli*

Pathway^a^	Medium^b^	Batch/fed-batch	Titer (g l^−1^)	Yield (g g^−1^)^c^	Productivity^d^	Comment	References
Thioesterase	TB	Batch	9.7	–	–		[25]
Thioesterase	Modified TB	Batch	4.4	0.49	0.18		[26]
Thioesterase	Minimal + YE	Batch Fed-batch	7.6 12.3	– 0.31	– 0.23	Aerated Aerated	[22]
Thioesterase	TB	Batch Fed-batch	4.3 7.20	0.43 –	– –		[20]
PTB/BUK; BCD	Minimal	Batch	0.3	–	–		[19]
CoA transferase	Modified TB	Batch Fed-batch	6.8 10.0	– –	– –	+ acetate	[23]
FA pathway + CoA transferase	LB	Batch	1.0	–	–		[24]
FA pathway + thioesterase	Minimal Minimal	Batch Fed-Batch	0.8 4.6	0.11 –	– –	Aerated Aerated	[21]
PTB/BUK^e^	Minimal + YE	Batch	33.1 ± 0.3	0.37 ± 0.01	0.89 ± 0.07		This study

–, Data not available

^a^Except for the FA (fatty acid) pathway, all other butyrate pathways utilized the basic *Clostridium* pathway with Ter substituting for BCD complex, unless stated otherwise, to butyryl-CoA followed by the indicated enzyme(s) for the conversion of butyryl-CoA to butyrate (Fig. 1). PTB, phosphotransbutyrylase; BUK, butyrate kinase; BCD, butyryl-CoA dehydrogenase complex

^b^TB, terrific broth; Minimal, mineral salt medium; YE, yeast extract; LB, Luria broth

^c^Yield—g butyrate.(g sugar consumed)^−1^

^d^Productivity—volumetric productivity is expressed as g l^−1^ h^−1^ and the values reported from this study are average with standard deviation over 24 h from three independent experiments

^e^Values from this study are from glucose- or xylose-mineral salt medium with yeast extract (5 g l^−1^) and are the average and standard deviation from three independent experiments. See text for details

## Results and discussion

### Construction of a butyrate-producing *E. coli*

Wild-type *E. coli* produces acetate, ethanol, formate, lactate, CO_2_ and H_2_ as fermentation products with small amount of succinate [29]. As a first step in the construction of a butyrate-producing microbial biocatalyst, the competing enzymes at the pyruvate node, lactate dehydrogenase (*ldhA*) and fumarate reductase (*frdABCD*) were removed (Fig. 1). Pyruvate formate–lyase served as the source of acetyl-CoA, the starting point of the introduced butyrate pathway (Fig. 1). To channel the acetyl-CoA to the butyrate pathway, *ackA*, and *adhE* were deleted (Fig. 1) and the resulting strain BEM9 was anaerobic growth negative due to its inability to oxidize the glycolysis-generated NADH and maintain redox balance. This deletion strain is expected to regain anaerobic growth upon introduction of the genes (*atoB, hbd, crt, ter, ptb* and *buk*) encoding enzymes that constitute the butyrate pathway (Fig. 1) since NADH oxidation by this pathway is designed to restore redox balance.

### Butyrate production by engineered *E. coli*

**Fig. 2:**
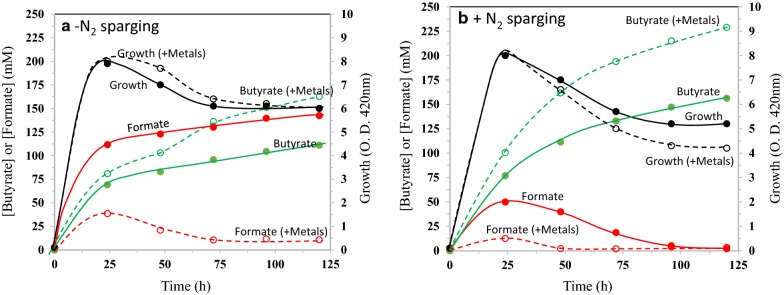
Effect of trace elements and N_2_-sparging on net formate concentration in the culture medium of *E. coli* strain LW393 and on butyrate production. Strain LW393 was cultured in LB-glucose (50 g l^−1^). Trace metals (Mo, Ni, Fe and Se) were added as indicated. See “Materials and methods” for other details. **a** Fermentations started with air in the gas phase. **b** N_2_ was sparged through the cultures at 7 ml min^−1^ throughout the experiment

In pH-controlled (7.0) batch fermentations in LB-glucose that started aerobically, strain LW393 carrying the genes encoding the butyrate pathway (Fig. 1) in two plasmids, pBEM3 (*atoB, hbd, crt* and *ter*) and p185 (*ptb* and *buk*), grew to an O. D. of 420 nm of about 8.0 and produced about 70 mM (~ 6 g l^−1^) butyrate in about 24 h (Fig. 2a). Butyrate concentration in the medium slowly increased to 110 mM (9.8 g l^−1^) at 120 h, a titer that is comparable to values reported in the literature for various recombinant *E. coli* strains in batch fermentation (Table 1) [19–26]. Formate was the major product of fermentation (110 mM at 24 h) and the molar ratio of formate to butyrate at 24 h was 1.6, a value that is close to the theoretical value of 2.0 (Fig. 1). As fermentation progressed, the formate/butyrate ratio decreased to 1.3 at 120 h, apparently due to FHL activity that removed part of the formate as CO_2_ and H_2_. Pyruvate and acetate were minor co-products and accounted for about 15% of the fermented glucose carbon, on a molar basis. These co-products lowered the butyrate yield to 0.4 g (g glucose consumed)^−1^. Presence of these co-products suggests that the rate of carbon flow from glucose to pyruvate and acetyl-CoA is higher than the rate of conversion of acetyl-CoA to butyrate.

### Effect of formate on butyrate production

**Fig. 3:**
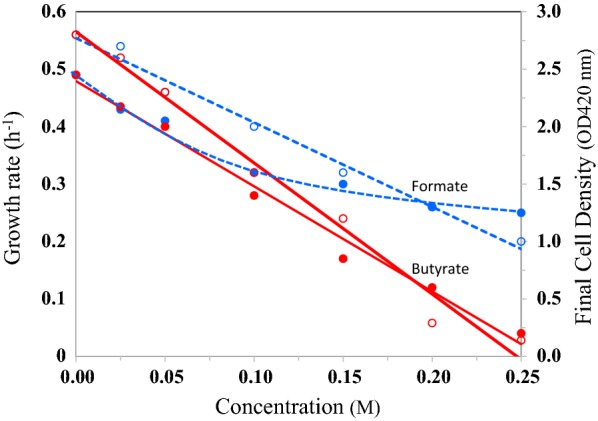
Effect of butyrate or formate on the growth and final cell density of *E. coli* strain LW393. Cultures were grown in LB + glucose (10 g l^−1^) + phosphate buffer (50 mM; pH 7.0) anaerobically at 37 °C with indicated concentrations of sodium butyrate or sodium formate. Final cell density was obtained after 25 h of incubation. Solid lines, final cell density; dashed lines, growth rate; solid symbols, growth rate; open symbols, final cell density

*Escherichia coli* formate dehydrogenase contains Mo, Se and non-heme Fe/S and the hydrogenase from this bacterium contains Ni and non-heme Fe/S. Thus, the production of active formate hydrogen–lyase complex requires Fe, Mo, Ni and Se. It is possible that LB medium is not providing these trace elements in support of optimal FHL activity and the accumulating formate is negatively impacting butyrate production. When the LB medium was supplemented with these trace metals, formate concentration was less than 40 mM at 24 h (compared to 110 mM in LB medium without trace metal supplementation) and at 120 h decreased to about 10 mM (Fig. 2a). This can be attributed to a 3.5-fold increase in FHL activity from 4.2 units (µmole h^−1^ mg cell dry weight^−1^) in LB-glucose medium to 14.7 units in the presence of added trace metals. Rapid removal of formate, an inhibitor of growth of *E. coli* also had a positive effect on butyrate titer (160 mM) (Fig. 2a; Fig. 3).

The butyrate titer of strain LW393 grown in LB-glucose with the four trace metals increased to 230 mM (20 g l^−1^) when N_2_ was sparged through the culture that maintained anaerobic condition (Fig. 2b). This significantly higher butyrate titer (compared to a culture that was not sparged with N_2_ and without trace metals; Fig. 2a) can be attributed to continued production of butyrate during the stationary phase of the culture. Sparging the culture with N_2_ also removed H_2_, the product of FHL and a known inhibitor of hydrogenase activity [30]. Under this fermentation condition, the formate concentration was only 10 mM at 24 h suggesting that the production of formate by pyruvate formate–lyase and removal by FHL are coupled. It is possible that the low formate concentration of the medium minimized the effect of formate inhibition as the culture entered stationary phase. Apparently, this lack of inhibition of metabolic activities of the culture by formate supported butyrate production until inhibition by accumulating butyrate is reached. Yield of butyrate in this fermentation condition was 0.45, higher than 90% of the theoretical yield of 0.49 g g^−1^ glucose fermented. Pyruvate and acetate accounted for the remaining glucose carbon (less than 7%). These results show that formate and its degradation products limit butyrate titer and yield of recombinant *E. coli*. Rapid removal of formate and H_2_ from the medium by the addition of trace metals and sparging with N_2_ appears to be an effective method to increase butyrate titer.

### Inhibition of growth by butyrate

It is interesting to note that the cell density of the butyrate-producing cultures started to decline during the stationary phase of growth (Fig. 2). It is possible that this decrease is associated with increasing butyrate concentration in the medium. Butyrate is reported to inhibit growth of *E. coli* and at pH 6.0, 11 mM butyrate inhibited growth by about 75% [31, 32]. Anaerobic growth of strain LW393 at pH 7.0 was also inhibited by about 50% by 0.1 M butyrate (Fig. 3). It is possible that growth and metabolic activity of *E. coli* are severely affected by accumulating butyrate and this inhibition also limits butyrate titer. It should be noted that although butyrate inhibited growth when added to the growth medium, in the fermentations described in this study, cultures were started without any added butyrate and the cultures reached late-exponential to early-stationary phase of growth before butyrate titer reached 0.1 M (Fig. 2).

### Butyrate titer in LB medium and energetics

**Fig. 4:**
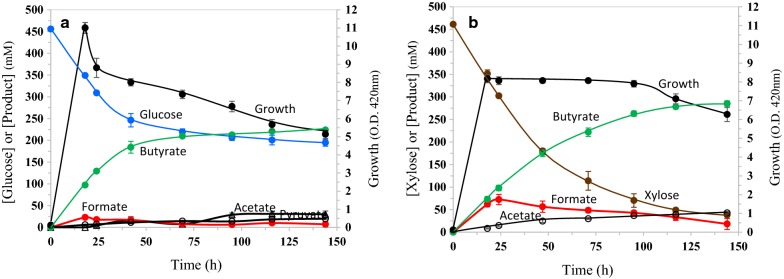
Fermentation profiles of *E. coli* strain LW393 in rich medium with glucose or xylose. Fermentations were in LB medium with trace elements and either glucose (**a**) or xylose (**b**) as the fermentable sugar. Culture temperature was 37 °C and the pH was maintained at 7.0. N_2_ was sparged through the culture to maintain anaerobic condition. Results are average of three independent experiments with standard deviation

The butyrate pathway used in this study with PTB/BUK generates three ATPs per glucose converted to butyrate (Fig. 1). With xylose as C source, the net ATP yield is 1.5 per xylose fermented to butyrate. On a comparable 6-carbon basis, the ATP yield is 1.8 ATP per xylose transported by the ABC transporter (XylFGH) compared to 3.0 per glucose transported by PTS system. Only about 50% of this ATP from xylose may be available to support growth and the remainder devoted to maintenance of cellular functions [33]. Lower ATP yield during sugar fermentation has been demonstrated to increase glycolytic flux and product titer [34, 35] and a similar increase in butyrate titer was also seen with xylose compared to glucose (Fig. 4; Table 2). As expected for a culture with higher ATP yield, glucose culture reached higher cell density than the xylose culture (ODmax of 11.0 ± 0.3 and 8.2 ± 0.2, respectively). Due to the higher cell density, butyrate titer was also higher at the end of 24 h for the glucose culture (130 ± 3 and 73 ± 6 mM, respectively, for glucose and xylose cultures). However, the specific productivity of butyrate for the glucose and xylose cultures was not different in this LB medium (Table 2). The advantage of xylose as C source in butyrate production is seen at the stationary phase during which the culture continued to ferment xylose to butyrate and the final butyrate titer was higher than that of the glucose culture (285 ± 6 and 220 ± 2 mM, respectively, for the xylose and glucose cultures). Growth in the LB+ xylose medium also negated the decline in cell density observed during the stationary phase of the glucose culture and is a putative reason for the observed higher butyrate productivity during the stationary phase of the LB-xylose culture (Fig. 4).

**Table 2:** Fermentation characteristics of *E. coli* strain LW393 and its derivatives

Strain	Relevant property^a^	Medium	Sugar	Cell density^b^	Butyrate titer (g l^−1^)	q_P_ (g g cells^−1^ h^−1^)^c^	Q_P_ (g l^−1^ h^−1^)^d^	Yield (g g^−1^ sugar)^e^
LW393	PTB/BUK	LB	Glucose	11.0 ± 0.03	19.7 ± 0.1	0.21 ± 0.01	0.47 ± 0.01	0.42 ± 0.01
		LB	Xylose	8.2 ± 0.03	25.1 ± 0.4	0.20 ± 0.03	0.36 ± 0.02	0.40 ± 0.01
		Mineral salts	Glucose	10.8 ± 0.20	28.4 ± 0.4	0.25 ± 0.04	0.64 ± 0.04	0.37 ± 0.01
		Mineral salts	Glucose + YE	10.5 ± 0.40	33.1 ± 0.3	0.26 ± 0.09	0.76 ± 0.04	0.35 ± 0.01
		Mineral salts	Xylose	9.4 ± 0.40	27.3 ± 0.8	0.30 ± 0.01	0.60 ± 0.02	0.37 ± 0.01
		Mineral salts	Xylose + YE	10.8 ± 0.17	31.7 ± 0.4	0.36 ± 0.03	0.89 ± 0.07	0.37 ± 0.01
LW483	(-PTB/BUK)	Mineral salts	Glucose	4.7 ± 0.04	14.6 ± 1.0	0.16 ± 0.02	0.20 ± 0.03	0.41 ± 0.01
LW532	LW483, Δ*tesB*	Mineral salts	Glucose	2.8 ± 0.20	8.8 ± 0.7	0.19 ± 0.03	0.12 ± 0.03	0.41 ± 0.02
LW612	LW532(p*tesB*)	Mineral salts	Glucose + (IPTG, 10 µM)	4.9 ± 0.10	20.1 ± 0.5	ND	0.30 ± 0.01	0.37 ± 0.01

Cultures were grown in LB or AM1 medium with glucose or xylose ± yeast extract (5 g l^−1^). All media were supplemented with additional trace elements as presented in “Materials and methods”. Fermentations were at 37 °C and the culture pH was maintained at 7.0 by automatic addition of 2 N KOH. N_2_ was sparged through the culture from the beginning (7 ml min^−1^) to maintain anaerobic condition

All reported values are average of three independent experiments with standard deviation

^a^(PTB/BUK), pathway from butyryl-CoA to butyrate utilizes phosphotransbutyrylase and butyrate kinase (Fig. 1). (-PTB/BUK), plasmid p185 encoding *ptb* and *buk* was removed from strain LW393. Strain LW612 carries plasmid pLW108 containing *E. coli tesB* expressed from *trc* promoter

^b^Cell density is presented as O. D. 420 nm

^c^q_P_, specific productivity; unit, g butyrate produced per g dry cell weight per h

^d^Q_P_, Average volumetric productivity of butyrate over a 24-h period

^e^Yield, g butyrate (g sugar consumed)^−1^

Although higher ATP yield is beneficial for growth, as seen with glucose as C source, the same higher ATP yield is apparently detrimental to butyrate production during stationary phase. In general, ATP produced during growth period is rapidly consumed by several reactions leading to biomass production and this sink for ATP is no longer available during the stationary phase. Absence of this coupling between ATP production by fermentation of sugars to butyrate and consumption by biosynthesis could be a reason for the observed decline in cell density and butyrate productivity in LB+ glucose medium. The physiological coupling between the net ATP yield and putative butyrate toxicity is unclear. The significantly lower ATP yield of the xylose culture, compared to glucose, is apparently supporting continued fermentation during the stationary phase (Fig. 4b).

### Production of butyrate in mineral salt medium

**Fig. 5:**
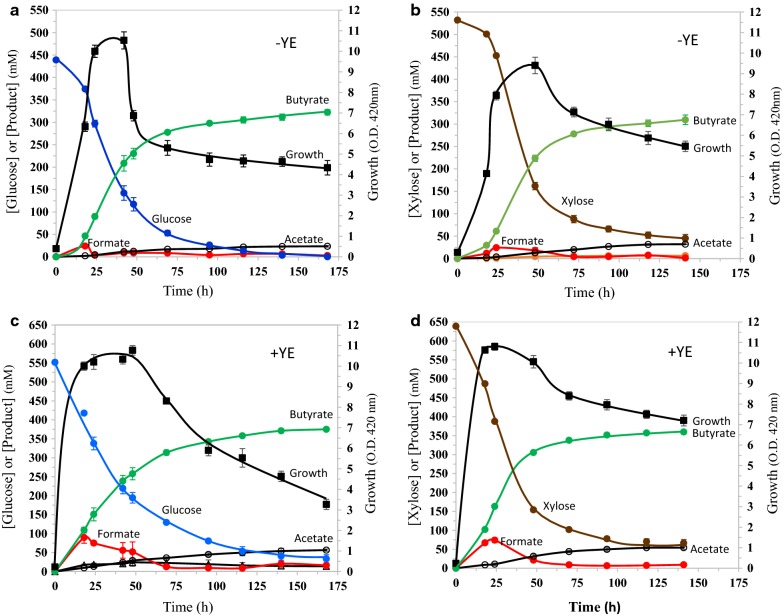
Fermentation of glucose or xylose to butyrate in mineral salt medium by *E. coli* strain LW393. Fermentations were in AM1 medium with additional trace metals at 37 °C and pH 7.0. N_2_ was sparged through the cultures at 7 ml min^−1^. **a** Glucose; **b** xylose; **c** glucose + yeast extract (5 g l^−1^); **d** xylose + yeast extract (5 g l^−1^). Other experimental conditions are the same as listed in Fig. 4

The differences seen between glucose and xylose fermentations in rich medium (Fig. 4) were negated by growing the cultures in mineral salt medium (Fig. 5a, b). This is apparently due to higher ATP demand of the cultures in a medium with limiting nutrients. Since converting sugars to butyrate is the only process that supports redox balance in strain LW393, the higher demand for ATP in mineral salt medium also increased both specific and volumetric butyrate productivity (Table 2). Strain LW393 grown in mineral salt medium with glucose yielded a butyrate titer of 323 ± 6 mM (28.4 g l^−1^), about 1.5-times higher than the value from the LB-glucose culture (220 ± 2 mM) (Figs. 4a, 5a). Butyrate accounted for 90% of the total products with acetate (24 mM) as the major co-product. This butyrate titer is close to three times higher than the highest butyrate titer reported in the literature for batch fermentations [25].

Butyrate titer in batch fermentation with glucose further increased to 375 ± 4 mM (33 g l^−1^) when yeast extract was included in the AM1 medium (Fig. 5c). This is the highest titer of butyric acid recorded for a recombinant *E. coli* producing butyrate and is comparable to the titer (32.5 g l^−1^) of *C. acetobutylicum* engineered for butyrate production [16]. Formate was not detectable in these cultures during the stationary phase due to the presence of trace metals and sparging with N_2_. These results further suggest the need for balance between energy production and consumption for supporting high product titer. Replacing glucose with xylose in mineral salt medium did not significantly change growth or fermentation characteristics of strain LW393 (Fig. 5b, d). In mineral salt medium, the cell density decreased during stationary phase in xylose medium also. Two factors could account for this decline in cell density: higher concentration of butyrate in the medium compared to rich medium and higher sensitivity of bacteria to inhibitors in mineral salt medium compared to rich medium (unpublished data).

Strain LW393 grown in mineral salt medium produced butyrate at a volumetric productivity that is significantly higher than the values calculated from fermentations in rich medium with either glucose or xylose (Table 2). The highest average Q_P_ value of 0.89 ± 0.07 g l^−1^ h^−1^ is comparable to the highest productivity values reported for *C. tyrobutyricum, C. thermobutyricum* or engineered *C. acetobutylicum* in batch fermentations (0.89–0.99 g l^−1^ h^−1^) [10, 14, 16, 36]. Further improvements in butyrate titer and productivity require *E. coli* derivates or other platform microorganisms that can tolerate higher butyrate concentrations than wild-type *E. coli*. These studies are in progress.

### Replacing PTB/BUK with thioesterases

Butyryl-CoA produced by the introduced pathway can be converted to butyrate by two pathways; an ATP-yielding PTB/BUK and an ATP-independent thioesterase (Fig. 1). The PTB/BUK pathway provides a net ATP yield of three per glucose fermented to butyrate. This ATP yield is 1.5 times higher than a thioesterase-based pathway and is expected to support higher growth rate, cell density and volumetric productivity. To evaluate this, plasmid p185 that carries the *ptb* and *buk* genes of *C. acetobutylicum* was removed from strain LW393 and the resulting strain LW482 did not grow anaerobically, apparently due to very low level of native thioesterase activity in this strain.

**Fig. 6:**
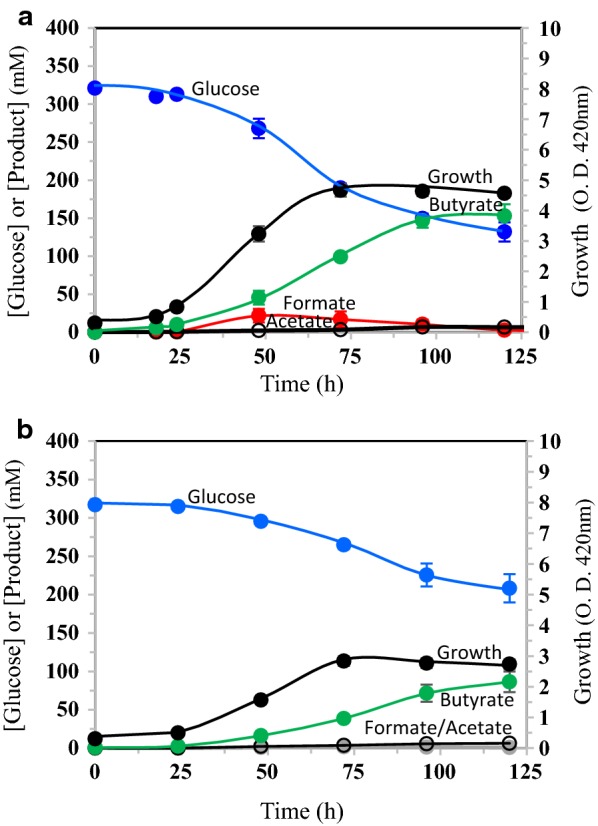
Thioesterase-based fermentation of glucose to butyrate by engineered *E. coli.* Fermentations were in mineral salt medium with glucose at 37 °C and pH 7.0 with N_2_ sparging. **a** Strain LW483; **b** strain LW532 (Δ*tesB*). Other experimental conditions are the same as listed in Fig. 4

Fermentation of glucose by strain LW482, in rich medium started with air in the gas phase, yielded less than 5 mM butyrate. Adaptive metabolic evolution for anaerobic growth in minimal medium yielded strain LW483 that grew at a lower growth rate in glucose-mineral salt medium (0.04 h^−1^) compared to strain LW393 (0.14 h^−1^) with PTB/BUK and the final cell density of the culture was about 50% of strain LW393 (Figs. 5a, 6a; Table 2). The highest butyrate titer produced by strain LW483 was also lower (165 ± 14 mM) compared to 323 ± 6 mM for strain LW393 with glucose. However, even this lower butyrate titer is still higher than the values reported in the literature for recombinant *E. coli* in batch fermentations (Table 1) [19–26]. Average volumetric productivity of butyrate for strain LW483 (TES) in AM1 medium was about 30% of the value for strain LW393 (PTB/BUK) (Table 2).

*E. coli* genome encodes multiple thioesterases and to identify the thioesterase that evolved to support butyrate production in strain LW483, five of the genes annotated to encode thioesterases (*tesA, tesB, paaI, ybgC* and *yciA*) along with their upstream DNA were cloned from the genomic DNA of strain LW483 and sequenced. Among these, only the sequence of *tesB* differed from that of *E. coli* W genome sequence by the presence of an IS4 family transposase within the coding region (inserted after amino acid 23). Eliminating the DNA downstream of this insertion (deletion of 194 amino acids; 49–242 of TesB) (strain LW532) lowered the cell density and butyrate titer by about 50% of the values for strain LW483 (Fig. 6b; Table 2). Average volumetric productivity of butyrate was also lower for strain LW532, 0.12 ± 0.03 g l^−1^ h^−1^, compared to 0.2 ± 0.03 g l^−1^ h^−1^ for strain LW483. Restoring TesB^+^ phenotype and increasing the copy number of *tesB* by introducing a plasmid (pLW108) with P*trc*-*tesB* into strain LW532 further increased butyrate titer and productivity (Table 2).

Apparently, TesB is one of the contributing thioesterases in addition to yet to be identified enzyme(s) that contribute to the removal of CoA from butyryl-CoA towards production of butyrate. Although Volker et al. identified YciA as the thioesterase that contributed to butyrate production in their recombinant *E. coli* [25], in strain LW483, the genome sequence of *yciA* was found to be the same as that of wild-type *E. coli* W and contribution of this enzyme to butyrate production in strain LW483 may be minimal. The 100 ± 10 mM (8.8 g l^−1^ at 180 h) butyrate produced by strain LW532 in batch fermentation is comparable to the reported butyrate titer of recombinant *E. coli* strains that utilize the thioesterase pathway (Table 1) [20, 22, 25, 26].

The enzyme(s) catalyzing the removal of CoA from butyryl-CoA in strain LW532 is yet to be identified. The absence of detectable change in the DNA sequence, including the promoter region, of four genes annotated as putative thioesterases (*tesA, paaI, ybgC* and *yciA*) suggests the presence of additional genes encoding this activity in the genome of *E. coli*. A mutation in *ackA* in strain LW532 also eliminates the potential of the *pta*/*ackA* pathway from evolving to catalyze the conversion of butyryl-CoA to butyrate. Whole genome sequencing followed by genetic analysis is expected to identify this yet to be characterized *E. coli-*encoded protein(s) that catalyzes this activity.

It is interesting to note that the specific butyrate productivity of strain LW483 utilizing thioesterase pathway was about 65% of the value for strain LW393 with the PTB/BUK pathway (Table 2). However, the lower cell density of strain LW483 compared to strain LW393 due to low ATP yield could only support a volumetric productivity that is 30% of the value for strain LW393. These results show that the additional energy generated by the PTB/BUK is essential to support higher growth rate and cell density of the microbial biocatalyst that in turn leads to higher volumetric productivity and butyrate titer.

## Conclusions

Metabolically engineered *E. coli* strains produced 375 ± 4 mM (33 g l^−1^) butyrate in batch fermentations in glucose-mineral salt medium supplemented with yeast extract (Fig. 5c). About 70% of this butyrate was produced during growth and early-stationary phase. Butyrate accounted for about 90% of the fermented glucose with acetate and pyruvate as co-products. Highest average volumetric productivity of butyrate was 0.89 ± 0.07 g l^−1^ h^−1^ in xylose-mineral salt medium with yeast extract. This butyrate titer and productivity are about three times higher than the literature values for butyrate-producing recombinant *E. coli* (Table 1). Lowering the ATP yield during stationary phase can further increase the titer of butyrate to levels that are comparable to Clostridia, the native butyrate producers.

## Materials and methods

### Materials

Organic and analytical-grade inorganic chemicals were from Fisher Scientific (Pittsburgh, PA). Biochemicals were from Sigma-Aldrich Co. (St. Louis, MO). Molecular biology reagents and supplies were from New England Biolabs (Ipswich, MA), Invitrogen (Carlsbad, CA), Clontech (Mountain view, CA), Zymo Research (Irvine, CA) or QIAGEN (Valencia, CA). DNA oligonucleotides were synthesized by Life Technologies (Carlsbad, CA).

### Bacterial strains and growth condition

*Escherichia coli* W (ATCC9637) served as the starting wild-type strain. A derivative of this strain with deletions in the following genes (Δ*adhE*, Δ*ackA*, Δ*ldhA*, Δ*frdABCD*) (strain BEM9) was constructed using standard genetic protocols [37, 38]. Strain BEM9 was used as the platform for engineering the butyrate pathway containing *atoB* from *E. coli*, *hbd, crt, ptb* and *buk* from *C. acetobutylicum* (ATCC 824), and *ter* from *Treponema denticola* (ATCC 35404). Based on DNA sequence, Ter from strain ATCC 35404 had two amino acid changes (D354E and S358G) compared to the enzyme from *T. denticola* ATCC 35405. The Ter protein from ATCC 35404 had a slightly higher specific activity (82 units; µmoles per min. mg protein) compared to the 73 units reported in the literature for the protein from *T. denticola* ATCC 35405 [18] when both proteins were expressed and purified from *E. coli*. Due to this higher specific activity, Ter from ATCC 35404 was used in this study. Strain LW393 carries the genes encoding the enzymes of the butyrate pathway in two plasmids, pBEM3 (*atoB, hbd, crt* and *ter*) and p185 (*ptb*, and *buk*) in strain BEM9 background. Bacterial strains and plasmids used in this study are listed in Additional file 1: Tables S1 and S2.

Cultures were grown in rich medium (LB) [39] or mineral salt medium (AM1) [40]. Both media were supplemented with additional trace elements to support formate hydrogen–lyase activity (per liter, FeSO_4_·7H_2_O, 10 mg; Na_2_MoO_4_·2H_2_O, 10 mg; NiCl_2_·6H_2_O, 1.18 mg; Na_2_SeO_3_, 0.263 mg). Betaine (1 mM) was added to AM1 medium when the sugar concentration exceeded 50 g l^−1^. Glucose or xylose served as C source for growth and fermentations. Yeast extract was added to AM1 medium at 5 g l^−1^, as needed.

Anaerobic cultures were grown in screw cap tubes filled to the top. Aerobic cultures were grown in Erlenmeyer flasks with medium at 10% of flask volume in a temperature-controlled shaker operating at 200 RPM. Clostridia were grown in reinforced clostridial medium (Oxoid, UK) in sealed 70-ml Wheaton bottles under N_2_. Fermentations were in 500-ml vessels with 250-ml medium with pH control using 2 N KOH as described previously [41]. These fermentations started aerobically with 250 ml of air in the gas phase. At a culture density of about 0.5 OD units (420 nm; 0.12 g cell dry weight l^−1^), O_2_ was not detectable in the medium and this O_2_ limitation led to production of fermentation products. Production of H_2_ and CO_2_ by FHL and release into the gas phase established anaerobic condition. As needed, N_2_ was passed through the culture at 7 ml min^−1^ to start and maintain fermentations under strict anaerobic condition. Late-exponential phase cultures grown with pH control at 7.0 served as inoculum for fermentations.

### Construction of butyrate pathway

The genes encoding the enzymes in the butyrate pathway were amplified by PCR and cloned into two plasmids. Promoterless *atoB* (from *E. coli*)*, hbd, crt* (from *C. acetobutylicum*) and *ter* (from *T. denticola*) in that order were cloned into plasmid pASG-IBA using Stargate procedure for constructing a synthetic operon as per the manufacturer’s protocol (IBA Gmbh, Gottingen. Germany) (plasmid pBEM3). Expression of these genes was found to be independent of *tet* promoter in the plasmid vector and thus, tetracycline or its analogs were not added to the medium. Using appropriate primers and *C. acetobutylicum* genomic DNA as template, *ptb* and *buk* including DNA 108 bp upstream of the *ptb* ORF and 80 bp downstream of *buk* ORF were amplified by PCR and cloned into plasmid vector pACYC184 (plasmid p185). Plasmids pBEM3 and p185 together provided the genes needed for the butyrate pathway from acetyl-CoA in *E. coli* (Fig. 1).

### Curing of plasmid p185 in strain LW393 (strain LW482)

Plasmid p185 in butyrate-producing *E. coli* strain LW393 was removed using CRISPR–Cas9 method described by Reisch and Prather [42]. Strain LW393 was transformed with plasmid pLW84 to induce Cas9 production and then transformed with pDC93 carrying phage λ Red genes and a DNA sequence for sgRNA (CCGGTGTCATTCCGCTGTTA). This DNA sequence is adjacent to a TGG sequence in the p15A origin of replication of plasmid p185 and is used for Cas9-based elimination of plasmid p185. Erythromycin- and spectinomycin-resistant colonies were selected on LB agar with the antibiotics. Several colonies were selected and cultured in LB containing 100 ng ml^−1^ anhydrotetracycline (aTc) at 30 °C for 2 h before plating on LB agar with spectinomycin and aTc. A chloramphenicol-sensitive colony, due to apparent loss of plasmid p185, was selected. Absence of plasmid p185 in this clone was confirmed by the lack of plasmid p185 in genomic DNA preparations as well as by the inability of the isolated genomic DNA to transform *E. coli* strain Top10 to chloramphenicol resistance. Plasmid pLW84 with Cas9 in the plasmid p185-cured cells was eliminated using the CRISPR method and plasmid pDC92-carrying sgRNA sequence (GCGCAGCGAGTCAGTGAGCG) and a temperature-sensitive replicon. After eliminating plasmid pLW84, plasmid pDC92 was removed by growing the culture at 37 °C. Strain LW482 contains only plasmid pBEM3 with *atoB, hbd, crt* and *ter* and its native plasmid pRK2 but not *ptb* and *buk*.

### Construction of strain LW532

Strain LW483, a spontaneous anaerobic growth-positive derivative of strain LW482 with butyrate as the fermentation product, carried an IS4-type transposase within the coding region of *tesB*. A 532-bp DNA within the coding region of *tesB* DNA downstream of the transposase was deleted from the genome of strain LW483 using the method described by Datsenko and Wanner [37]. A kanamycin-resistant transformant was selected and the deletion within the *tesB* coding region was verified by PCR and DNA sequencing (strain LW532).

### Construction of plasmid pLW108

Plasmid pLW108 carries *E. coli tesB* under the *trc* promoter. Native *tesB* without the promoter was amplified from the genomic DNA by PCR using appropriate primers. The PCR product was cloned into plasmid vector pTrc99a using the SLIC method [43]. After transformation, plasmid containing derivatives of *E. coli* strain Top10 were selected as ampicillin-resistant colonies and the presence of *tesB* was confirmed by DNA sequencing.

### Analytical methods

Cell density of the cultures was determined as optical density at 420 nm using Beckman DU640 spectrophotometer (Indianapolis, IN). Sugars and organic acids were determined by HPLC as described previously [44].

## Additional file

**Additional file 1: Table S1.** *E. coli* strains constructed and used in this study. **Table S2**. *E. coli* plasmids constructed and used in this study.
