# Supplementary material for: Metabolic engineering of Escherichia coli for the production of butyric acid at high titer and productivity
Source: Biotechnol Biofuels. 2019 Mar 22;12:62. doi: 10.1186/s13068-019-1408-9 (PMC6429758; doi:10.1186/s13068-019-1408-9)
Supplement: Supplementary file 1 — Additional file 1: Table S1. E. coli strains constructed and used in this study. Table S2. E. coli plasmids constructed and used in this study. [file 13068_2019_1408_MOESM1_ESM.docx]

Metabolic engineering of *Escherichia coli* for production of butyric acid

Liang Wang^1^, Diane Chauliac^1,3^, Brelan E. Moritz^1,4^, Guimin Zhang^1,2^, Lonnie O. Ingram^1^ and K. T. Shanmugam^1^*

^1^Department of Microbiology and Cell Science,

University of Florida, Gainesville, Florida 32611, USA

^2^State Key Laboratory of Biocatalysis and enzyme engineering,

College of Life Science, Hubei University, Wuhan 430062, China.

Supplementary Information

Table S1. *E. coli* strains constructed and used in this study

Strain Relevant genotype Source or Reference

*E. coli* W (ATCC9637) Wild type ATCC

SE2274 W, Δ*ldhA* Lab collection

BEM3 SE2274, Δ*adhE* This study

BEM9 BEM3, Δ*ackA*, Δ*ldhA*, *frdA*::IS This study

LW310 BEM9, pBEM3, p185 This study

LW393 LW310, Δ*frdABCD* This study

LW483 LW393, (Δp185) This study

LW523 LW483, p185 This study

LW532 LW483, Δ*tesB*  This study

LW612 LW532, pLW108 This study

*Clostridium acetobutylicum* ATCC 824 USDA-ARS-NRRL

*Treponema denticola* ATCC 35404 L. Kesavalu

Wild type *E. coli* W was from ATCC. *C. acetobutylicum* was obtained from ARS (NRRL) culture collection, Peoria, IL. *T. denticola* genomic DNA was provided by Dr. K. Lakshmyya, University of Florida. All other strains were constructed during this study.

Table S2. *E. coli* plasmids constructed and used in this study

Plasmid Relevant genotype Source/Reference

pASG-IBA ColEI, *tetR*, Ap^R^ IBA-Gmbh

pBEM3 pASG-IBA, P*tet*-*atoB-_Ec_, (hbd, crt)-_Ca_, ter-_Td_* This study

p185 pACYC184, *ptb, buk* (*C. acetobutylicum*) This study

pE194 Em^R^ D. Dubnau

pRK2 native plasmid of *E. coli* W, 5360 bp [1]

pKD4 Ap^R^, FRT-Km-FRT B. Wanner

pKDsg-ack Ptet-sgRNA-*ack*, P*araB*-λRed Addgene

pCas9CR4 Ptet-*cas*9, *tetR*, Cm^R^ Addgene

pDC74 pRK2, FRT-Km-FRT (pKD4) This study

pDC83 pDC74, zeo^R^ This study

pDC87R pRK2, Cas9, zeo^R^ This study

pDC92 pKDsg-pRK2 This study

pDC93 pKDsg-pCas9 This study

pDC95 pRK2-cas9-cr2, zeo^R^ This study

pLW84 pDC95, Em^R^ (pE194) This study

pLW108 pTrc99a, *tesB* This study

**Reference**

1. Turner PC, Yomano LP, Jarboe LR, York SW, Baggett CL, Moritz BE, Zentz EB, Shanmugam KT, Ingram LO: Optical mapping and sequencing of the *Escherichia coli* KO11 genome reveal extensive chromosomal rearrangements, and multiple tandem copies of the *Zymomonas mobilis pdc* and *adhB* genes. *J Ind Microbiol Biotechnol* 2012, 39:629-639.
